# A strategy for the generation, characterization and distribution of animal models by The Michael J. Fox Foundation for Parkinson’s Research

**DOI:** 10.1242/dmm.011940

**Published:** 2013-09-12

**Authors:** Marco A. S. Baptista, Kuldip D. Dave, Niketa P. Sheth, Shehan N. De Silva, Kirsten M. Carlson, Yasmin N. Aziz, Brian K. Fiske, Todd B. Sherer, Mark A. Frasier

**Affiliations:** 1 The Michael J. Fox Foundation for Parkinson’s Research, New York, NY 10018-6798, USA

## Abstract

Progress in Parkinson’s disease (PD) research and therapeutic development is hindered by many challenges, including a need for robust preclinical animal models. Limited availability of these tools is due to technical hurdles, patent issues, licensing restrictions and the high costs associated with generating and distributing these animal models. Furthermore, the lack of standardization of phenotypic characterization and use of varying methodologies has made it difficult to compare outcome measures across laboratories. In response, The Michael J. Fox Foundation for Parkinson’s Research (MJFF) is directly sponsoring the generation, characterization and distribution of preclinical rodent models, enabling increased access to these crucial tools in order to accelerate PD research. To date, MJFF has initiated and funded the generation of 30 different models, which include transgenic or knockout models of PD-relevant genes such as *Park1* (also known as *Park4* and *SNCA*), *Park8* (*LRRK2*), *Park7* (*DJ-1*), *Park6* (*PINK1*), *Park2* (*Parkin*), *VPS35*, *EiF4G1* and *GBA*. The phenotypic characterization of these animals is performed in a uniform and streamlined manner at independent contract research organizations. Finally, MJFF created a central repository at The Jackson Laboratory (JAX) that houses both non-MJFF and MJFF-generated preclinical animal models. Funding from MJFF, which subsidizes the costs involved in transfer, rederivation and colony expansion, has directly resulted in over 2500 rodents being distributed to the PD community for research use.

## Introduction

Parkinson’s disease (PD) is the second most common neurodegenerative disease and is characterized by both motor and non-motor deficits (Lees et al., 2009). The neuropathology consists of loss of dopaminergic (DA) cells in the substantia nigra, decreased DA neurotransmission, and the accumulation of cytoplasmic inclusions called Lewy bodies, containing insoluble α-synuclein protein, in multiple cell types (Bernheimer et al., 1973; Fearnley and Lees, 1991; Jellinger, 2009). The clinical motor manifestations of PD include bradykinesia, rigidity and resting tremors. Non-motor symptoms such as olfactory deficits, sleep disturbances and cognitive dysfunction are also recognized as important symptoms but their respective pathophysiologies are less understood (Weintraub et al., 2004). There is an urgent unmet clinical need to improve current treatment strategies, which can be associated with serious side effects (e.g. L-dopa-induced dyskinesias), and to provide new neuroprotective and/or disease-modifying therapies (Meissner et al., 2011).

Animal models are crucial for understanding disease mechanisms and guiding subsequent drug discovery. Predictive validity in animal models establishes confidence that an emerging therapy will be successful in the clinic. However, it has been a challenge to develop models that fully recapitulate the main features of PD. The first animal models developed for PD made use of neurotoxins that selectively lesion specific neurons. Toxins such as 6-hydroxydopamine (6-OHDA), 1-methyl-4-phenyl-1,2,3,6-tetrahydropyridine (MPTP), reserpine, rotenone and paraquat have been effective in acutely inducing DA cell loss; however, these do not seem to drive the progressive neurodegeneration that is evident in PD (Betarbet et al., 2000; Brooks et al., 1999; Carlsson et al., 1957; Heikkila et al., 1985; Przedborski et al., 2001). Also, these neurotoxins do not induce the accumulation of Lewy body inclusions, which is a hallmark of PD in humans. Furthermore, the complex progressive dysfunction and interplay of neural pathways that impact motor and non-motor symptoms of PD are not addressed by toxin approaches. For example, in humans, sleep, olfactory and gastrointestinal disturbances precede DA-associated motor disturbances by many years, and certain motor deficits such as freezing (temporary, involuntary inability to move) are not responsive to DA therapy so are not likely to be related to dopamine (Lees et al., 2009). The Michael J. Fox Foundation (MJFF) is the largest private funder of Parkinson’s disease research and has funded preclinical and clinical studies worldwide. Recently, MJFF has focused on the generation of novel genetically engineered rodent models to overcome the limitations inherent in using neurotoxins to generate models of this disease. It is hoped that this endeavor will accelerate preclinical research in PD.

New and improved animal models of PD are required to better understand the neuropathophysiology of the disease. Genetic factors have been increasingly associated with PD (Simón-Sánchez et al., 2009); however, so far the implicated loci explain only a small percentage of cases. Nonetheless, studying rare pathological mutations could point to the biology underlying idiopathic forms of the disease. Mutations and/or variation in α-synuclein (encoded by *SNCA*; also known as *Park1* and *Park4*), leucine rich-repeat kinase 2 (*LRRK2*; also known as *Park8*), phosphatase and tensin homolog (PTEN)-induced putative kinase 1 (*PINK1*; also known as *Park6*), *Parkin* (also known as *Park2*), *DJ-1* (also known as *Park7*), glucocerebrosidase (*GBA*), vacuolar protein sorting-associated protein 35 (*VPS35*) and eukaryotic translation initiation factor 4 gamma 1 (*EIF4G1*) have all been implicated in familial and sporadic PD (Houlden and Singleton, 2012). The generation of models based on these genetic discoveries will provide crucial tools for understanding the pathophysiology of PD and provide preclinical models to test future therapeutics.

In 2005, MJFF sponsored a workshop with the goal of addressing the lack of standard models that reproduce the progressive and degenerative phenotype of the disease; such models are crucial for developing neuroprotective therapies. As an outcome, MJFF launched an initiative to generate or characterize genetic and toxin models of PD, and funded seven rodent model projects (Cannon et al., 2013; Daher et al., 2009; Ekstrand et al., 2007; Frank-Cannon et al., 2008; Kuo et al., 2010; Sotiriou et al., 2010; Tong et al., 2009). Although the program successfully generated animal models that reproduce certain PD-like phenotypes, licensing restrictions limited the availability of many of these models to the PD research community. Thus, to widen access, MJFF moved to a strategy of generating its own animal models and to facilitate distribution through an independent repository. A steering committee of leading PD researchers (see Acknowledgements) was formed to guide MJFF in its efforts.

This approach has allowed MJFF to establish new collaborative opportunities. For example, in 2010, MJFF launched a program in collaboration with Elan Pharmaceuticals to generate seven mouse models against diverse PD targets, including *SNCA*, *EIF4G1*, *VPS35* and *GBA* (see Table 1). The program included a multistage approach to characterizing animal models. Initial data included quality assurance such as gene expression levels, integration sites, protein expression and heritability. A second phase included obtaining a minimum set of phenotypic data in aged cohorts: behavioral outcome measures, striatal neurochemistry and substantia nigra stereology. MJFF and Elan Pharmaceuticals also developed a material-transfer agreement that allowed for broad distribution to both academic and industrial institutions.

**Table 1.**
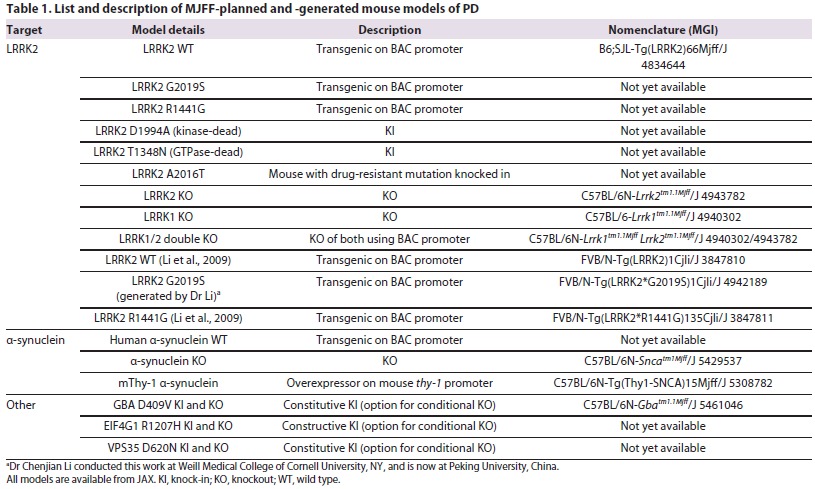
List and description of MJFF-planned and -generated mouse models of PD

To date, MJFF has invested substantially in building its preclinical animal models portfolio, concentrating on three strategies: generating new models, standardizing phenotypic characterization of new and existing models, and increasing distribution. MJFF has funded the development of 30 different models in an effort to make them available at low cost with limited restrictions on use for both academia and industry. The models include transgenic or knockout models of PD-relevant genes such as *LRRK2*, *SNCA*, *DJ-1*, *PINK1*, *Parkin*, *VPS35*, *EIF4G1* and *GBA*. A lack of standardization and varying methodologies has made it difficult to compare outcome measures across laboratories (Brown and Moore, 2012). For example, factors introduced in the everyday laboratory environment (e.g. undue stress from unfamiliar odors stemming from using multiple lab coats during an experiment) and the type of behavioral tasks chosen (exploratory versus learned or innate skill tests) might influence the results of a study (Potashkin et al., 2010). By sponsoring a standardized comparison of new and existing preclinical models at independent laboratories, we have taken a proactive approach to streamline phenotypic characterization and develop a uniform process. Rodents are grown to 4, 8 or 12 months of age, and undergo behavioral, neurochemical and pathological characterization to determine whether they exhibit a PD-like phenotype. Over 30 different central nervous system (CNS) and non-CNS tissues are collected, stored and made available to researchers for further detailed characterization. Animals are ultimately transferred to The Jackson Laboratory (JAX) for re-derivation and colony expansion. This has directly resulted in increased use of these mouse models in the research and drug-discovery communities (e.g. Cannon et al., 2013; Ness et al., 2013). Here, we describe the MJFF strategy of generating, characterizing and distributing rodent models, and explore the many ways that these models could be used to promote PD research and drug development.

## Generation of animal models

**Fig. 1.**
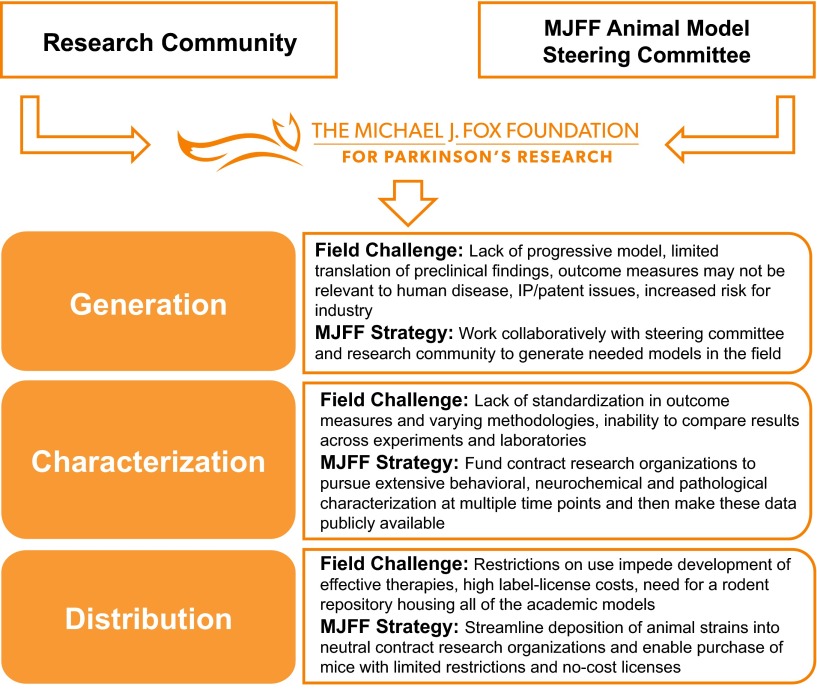
**MJFF**′**s strategy to address challenges in generating, characterizing and distributing animal models of Parkinson’s disease.**

As outlined above, a challenge in animal model generation facing the PD field is the lack of existing genetic models that closely recapitulate the PD-related CNS pathology. The MJFF strategy has fostered collaborations with the research community to generate animal models needed to advance the PD field (see Fig. 1). MJFF focuses on developing genetically engineered mice and rats based on targets that have been associated with PD through human genetic studies. Human genetics provide an insight into the mechanisms affected in sporadic idiopathic PD, and animal models based on these associations might reproduce the features of PD. One challenge has been to prioritize the animal models being generated. Relying on evidence generated by the research community, we focused on some of the most common genetic causes of the disease. To date, we have funded the generation of 30 transgenic or knockout rodent models of PD (18 mouse and 12 rat models), with a focus on the *LRRK2*, *SNCA*, *PINK1*, *DJ-1*, *Parkin, GBA*, *VPS35* and *EIF4G1* genes (Tables 1, 2). These models are described in detail below. All animal work in these studies is in compliance with the National Institutes of Health (NIH) policy on humane animal welfare and has been approved by the Taconic, JAX, Ozgene, WIL Research, SAGE and Psychogenics Institutional Animal Care and Use Committees (IACUCs).

**Table 2.**
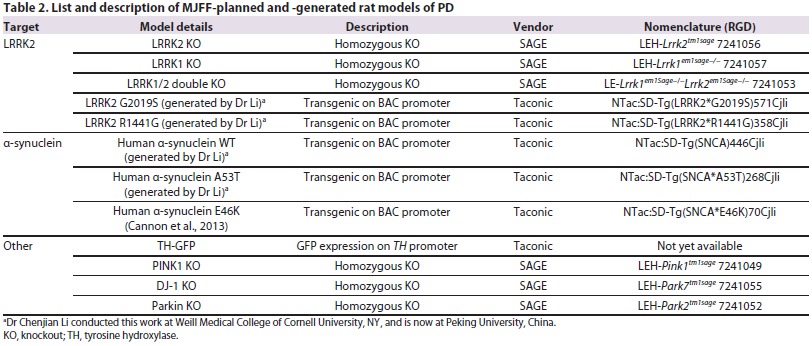
List and description of MJFF-planned and -generated rat models of PD

## Leucine rich-repeat kinase 2 (*LRRK2*) models

The autosomal-dominant *Park8* locus was linked to PD in 2002, and was followed by discoveries in 2004 linking missense mutations in the gene (renamed *LRRK2*) to familial PD (Funayama et al., 2002; Zimprich et al., 2004). Since then, five *LRRK2* mutations (G2019S, R1441G, R1441C, Y1699C and I2020T) have been unequivocally linked to both familial and sporadic forms of the disease (Melrose, 2008). The pathological mutations are located predominately in the enzymatic region of LRRK2, with the most common form, G2019S, increasing kinase activity of the protein (West et al., 2005). Because the phenotype of *LRRK2* mutation carriers seems to be indistinguishable from that of individuals with idiopathic PD (Marras et al., 2011), it has been hypothesized that targeting the increased LRRK2 kinase activity caused by these mutations might not only be therapeutic to those carrying the mutation but also to those who have the idiopathic form of the disease. An *LRRK2* animal model that recapitulates PD features is a crucial tool that is needed for both academia and industry. In light of the strong link between *LRRK2* and PD, in addition to the availability of a myriad of kinase inhibitors in chemical libraries from oncology programs, many pharmaceutical companies have LRRK2 kinase inhibitor programs. Industry needs an *LRRK2* animal model with a consistent and robust phenotype to determine both the efficacy of putative drugs and the window of safety.

The challenge has been to develop a robust *in vivo* model to test these LRRK2 kinase inhibitors. All of the *LRRK2* mutant animal models to date do not completely recapitulate the hallmarks of PD (i.e. DA neuronal loss, Lewy bodies and a behavioral phenotype). For example, the *LRRK2* R1441G mutant mouse displays diminished dopamine release and axonal pathology of nigrostriatal DA projections without neuronal cell loss (Li et al., 2009). Furthermore, it has recently been discovered that the loss of LRRK2 kinase activity and/or protein levels induces a pathological phenotype in kidney and lung tissues (these tissues express a high level of LRRK2) (Herzig et al., 2011; Tong et al., 2010). To address these challenges, MJFF has generated the following mutants (both mice and rats): those with pathological mutations in the kinase or GTPase domains (G2019S and R1441G, respectively) of LRRK2; expressing kinase- or GTPase-dead mutants (D1994A and T1348N, respectively); with *LRRK1* or *LRRK2* knocked out; and expressing an LRRK2 kinase-inhibitor drug-resistant mutant (A2016T) (see Table 1). By creating different *LRRK2* mutants using different genetic strategies [bacterial artificial chromosome (BAC), knock-in, zinc finger nuclease (ZFN) knockout] and different rodent species, MJFF is attempting to increase the probability of creating an animal model that will be useful for both understanding *LRRK2* biology and in a drug discovery screen.

## Alpha-synuclein (*SNCA*) models

In 1997, the A53T mutation in the *SNCA* gene was identified in a familial case of early-onset PD (Polymeropoulos et al., 1997). Since then, two other missense mutations (A30P and E46K) as well as duplication and triplication of the *SNCA* gene locus have been linked with the disease (Chartier-Harlin et al., 2004; Krüger et al., 1998; Singleton et al., 2003; Zarranz et al., 2004). The SNCA protein is expressed in the substantia nigra DA neurons, enriched in Lewy bodies, and linked to both the etiology and pathogenesis of PD (Iwai et al., 1995). The protein is located primarily in the presynaptic vesicles (Iwai et al., 1995) and might be important in neurotransmitter release (Murphy et al., 2000). However, there are currently many gaps in the scientific literature surrounding the normal function of *SNCA*, there is a lack of an *in vivo* marker to monitor disease progression and therapeutic efficacy, and there is no clear strategy of how to target SNCA in the clinic. Furthermore, there is no transgenic SNCA model that recapitulates all of the features of progressive PD pathology. In order to introduce more widely available tools to probe these issues in the *SNCA* field, MJFF has generated: a wild-type human *SNCA*-expressing strain (a much-needed control in the research field), an mThy-1 *SNCA* strain (to overexpress SNCA protein in the brain, including in the nigrostriatal pathway), and a knockout mouse (see Tables 1 and 2) (Fleming et al., 2006; Rockenstein et al., 2002). In generating these multiple *SNCA* animal models, MJFF hopes to provide the research community with the tools to develop a better therapeutic against pathological *SNCA*.

## PTEN-induced putative kinase 1 (*PINK1*) models

Although *PINK1* mutations are extremely rare, the emerging understanding of *PINK1* function could elucidate a common mechanism of pathology in idiopathic PD. *PINK1* is a mitochondrial protein that is located in the matrix and the intermembrane space, and is ubiquitously expressed in the brain (Silvestri et al., 2005). Evidence suggests that the G309D mutation impairs the normal protective effect of wild-type *PINK1* on mitochondria by interfering with adenosine diphosphate binding (Valente et al., 2004). Knockout models of the *Drosophila PINK1* ortholog have defective mitochondrial morphology and are susceptible to oxidative stress (Clark et al., 2006). Given the evidence that *PINK1* deficiency causes mitochondrial dysfunction, MJFF has generated a homozygous *PINK1* knockout rat by deleting 26 base pairs in exon 4 using the SAGE ZFN technology. Although *PINK1* knockout mouse models have been generated (Gispert et al., 2009), the *PINK1* ZFN knockout rat, which displays a PD-like phenotype (our unpublished data, in preparation), can be used to test *PINK1* therapeutics in a different species.

## *DJ-1* models

Recent studies suggest that the DJ-1 protein plays an important role in sporadic late-onset PD. However, very few individuals with *DJ-1* mutations have been identified and thus little is known about the effects of such mutations (Dekker et al., 2004). DJ-1 is a cytoplasmic protein that can also translocate into the mitochondria and seems to act as an antioxidant (Zhang et al., 2005). The antioxidant function of *DJ-1* could be particularly important in nigral dopamine neurons, which are exposed to particularly high levels of oxidative stress. In brains from individuals with sporadic PD, greater oxidative damage of DJ-1 and a significant increase in total DJ-1 protein levels have been observed, compared with normal controls (Choi et al., 2006). Mutant DJ-1 seems to interact with Parkin (described below), whereby Parkin acts as an E3 ligase to remove mutated DJ-1 (Moore et al., 2005). *DJ-1*-null mice are hypersensitive to oxidative stress and MPTP (Kim et al., 2005). Although there are *DJ-1* knockout mice available, the research community would benefit from having another species with *DJ-1* deficiency to be able to conduct other types of experiments (e.g. 6-OHDA lesions) that are normally conducted in rats. Therefore, MJFF has employed ZFN technology to knock out *DJ-1* in the rat and generate an animal model that exhibits gait and locomotor deficits (our unpublished data, in preparation).

## Parkin model

The *Parkin* gene was first linked to autosomal-recessive juvenile PD in 2008, and several mutations in the *Parkin* gene have now been associated with early-onset PD (Hattori et al., 1998; Kitada et al., 1998). *Parkin*-deficient mouse models of PD do not display loss of DA neurons (Goldberg et al., 2003) but do exhibit elevated extracellular dopamine levels (Goldberg et al., 2003), enhanced dopamine metabolism (Itier et al., 2003) and indications of an impaired respiratory capacity of striatal mitochondria (Palacino et al., 2004). MJFF again used the SAGE ZFN technology to knock out *Parkin* in the rat in order to determine whether there are species-specific differences between rodents. However, preliminary analyses indicate that Parkin KO rats have a normal phenotype up to 12 months of age (our unpublished data, in preparation).

## Glucocerebrosidase (*GBA*) models

Small subsets of individuals with Gaucher’s disease (caused by a defect in the *GBA* gene, which encodes for glucocerebrosidase) develop parkinsonian symptoms, including tremor, rigidity and bradykinesia (Bembi et al., 2003; Machaczka et al., 1999; Neudorfer et al., 1996). Analysis of post-mortem brain tissue revealed that some such individuals had cortical Lewy bodies corresponding to Braak stages 5–6, in addition to the classic PD pathology (Neumann et al., 2009), although a subsequent study has found that *GBA* mutations do not exacerbate the Lewy body phenotype (Parkkinen et al., 2011). Therefore, it has been proposed that mutations in the *GBA* gene might be a risk factor for the development of PD (Lwin et al., 2004). In an analysis of brains from 57 subjects with PD, it was found that 12 samples displayed alterations in *GBA*, which included mutations (N370S, L444P, K198T and R329C) and probable polymorphisms (T369M and E326K) (Lwin et al., 2004). Recently, it has been reported that *GBA* mutations promote the accumulation of *SNCA* in a dose- and time-dependent manner (Cullen et al., 2011). In mice expressing two D409V mutant knock-in alleles, an age-dependent rise of endogenous *SNCA* in hippocampal membranes was detected at 52 weeks of age. The authors of this study proposed a possible role for autophagy in mediating this effect, because rapamycin reverses this accumulation of *SNCA*, at least in cell culture (Cullen et al., 2011). Therefore, MJFF has generated a D409V knock-in and knockout mouse that is available at JAX (see Table 1) that could be used to probe how GBA mutations affect SNCA processing and increase the risk of PD.

## Vacuolar protein sorting-associated protein 35 (*VPS35*) and eukaryotic translation initiation factor 4 gamma (*EIF4G1*) models

Both the *VPS35* and *EIF4G1* mutations are rare with autosomal-dominant inheritance. They have only been identified in a few families (Chartier-Harlin et al., 2009; Vilariño-Güell et al., 2011; Wider et al., 2008; Zimprich et al., 2011). The associated disease phenotype is consistent with levodopa-responsive PD and some cases of dementia (Fujioka and Wszolek, 2012). Although the pathophysiology of mutant *EIF4G1* (encoding a component of EIF4G that is involved in the recognition of mRNA-cap-dependent translation) is linked to *SNCA*, the relationship between *SNCA* and *VPS35* is unknown (Fujioka and Wszolek, 2012). Recently, it has been reported that PD-associated defects in *RAB7L1* or *LRRK2* lead to a deficiency of the VPS35 component of the retromer complex. Expression of wild-type *VPS35* rescued these defects (MacLeod et al., 2013). By generating animal models that have these mutations, MJFF is helping to contribute tools that will further elucidate the pathophysiology of these newly discovered PD-linked gene mutations.

## Characterization

**Fig. 2.**
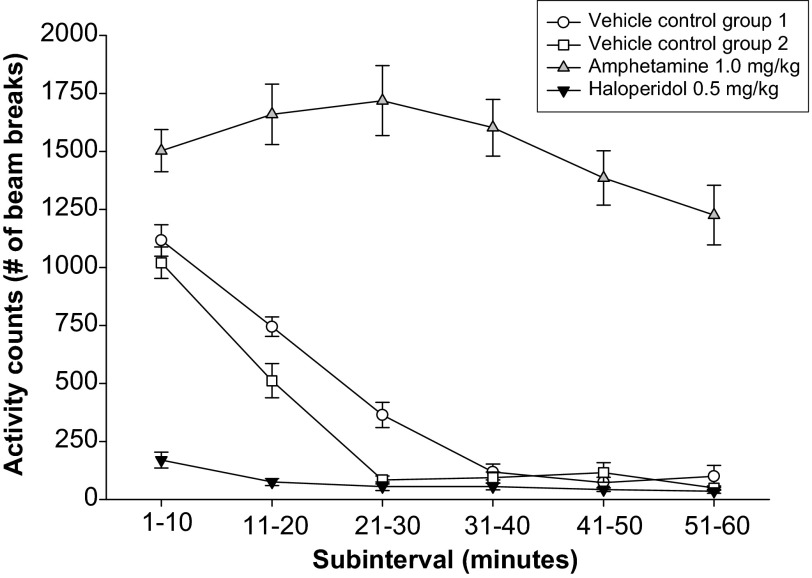
**Motor activity was assessed in Sprague-Dawley rats (similar data obtained with CD-1 mice) by number of beam breaks.** When rats pass through infrared beams it is recorded as a beam break. Two separate vehicle control groups reveal similar activity counts across the 60 minutes. Amphetamine (1.0 mg/kg) and haloperidol (0.5 mg/kg) were used to provide a positive and negative control, respectively, to further validate the assay. These drugs are common controls that are expected to increase (amphetamine) or decrease (haloperidol) motor activity. Data were obtained from WIL Research validation efforts.

Many labs have characterized animal models using different methods. This lack of standardized data makes it difficult to evaluate both the relevant phenotypes and the robustness of an animal model. In an attempt to address the lack of standardization in outcome measures and the use of varying methodologies when examining animal models, MJFF partnered with contract research organizations (CROs) to pursue an extensive and systematic behavioral, neurochemical and pathological characterization of models. A detailed list of the different phenotypes being measured can be seen in Table 3. Behavior, neurochemistry and pathology are being examined in 4-, 8- and 12-month-old animals by different research institutions (WIL Research, Psychogenics and Neuroscience Associates). Behavioral, neurochemical and stereological assays have also been validated at these respective CROs [see Fig. 2 for behavioral assay validation based on data from WIL Research; Quang et al. for neurochemistry (Quang et al., 2012); Healy-Stoffel et al. for stereology (Healy-Stoffel et al., 2012)]. Recognizing the importance of *SNCA* and *LRRK2* transgenic animals, MJFF initiated a standardized characterization of these models in order to formulate a best practices guide for the research community.

**Table 3.**
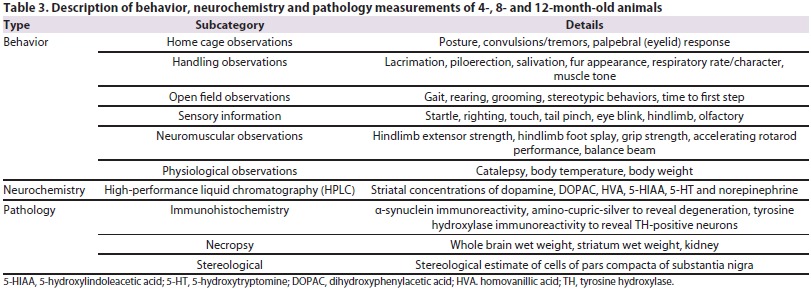
Description of behavior, neurochemistry and pathology measurements of 4-, 8- and 12-month-old animals

As part of the joint MJFF–Elan-Pharmaceuticals animal models program, MJFF initiated a systematic analysis of five *SNCA* mouse models (Giasson et al., 2002; Hilton et al., 2010; Kuo et al., 2010; Prasad et al., 2011; Rockenstein et al., 2002). All five models show *SNCA* aggregation or accumulation in the brain, as well as other PD-related phenotypes (Magen and Chesselet, 2010). However, these models were characterized in different laboratories with varying behavioral and neurochemical outcome measures. Also, MJFF initiated a systematic characterization of behavior, neurochemistry and pathology of *LRRK2* G2019S mice, *LRRK2* R1441G mice and *LRRK2* knockout rats to develop a consistent dataset on all of these animals. The goal is to build a consensus on what phenotypic features the animal models display so that they can be employed by PD researchers and potentially be used for drug screening.

## Distribution

Researchers currently face many restrictions on the use of animal models, which impedes the development of effective therapies for PD. Typically, the distribution of animal models occurs either through direct collaboration by investigators or investigators depositing their mice in a repository. Industry researchers also encounter challenges due to high licensing fees for access to these animals. The philosophy of MJFF has been to reduce the barrier of access to animal models for both industry and academia. As a result, we have three distinct distribution initiatives: MJFF-funded and -generated models, MJFF-funded and investigator-generated models, and MJFF-supported models. In the first initiative, MJFF drives the entire generation of the animal model through institutions or CROs to create the model. These 22 models are available at JAX, Taconic and SAGE (Tables 1, 2). An example is the *LRRK2* T1348N GTPase-dead mouse that was generated and characterized via an MJFF-led effort to investigate the role of the enzymatic domain of LRRK2 on the function of this protein. In the second approach, MJFF works with PD researchers with expertise in creating and distributing animal models. MJFF actively requests that these investigators deposit their models into rodent repositories. To facilitate this, MJFF subsidizes the costs of transferring animals to the repository, rederivation, colony expansion and housing for eventual distribution of these models. MJFF has facilitated the importation of over 25 PD strains from academic labs to rodent repositories, with over 2500 rodents being distributed out to the research community, and with 16 live and 11 cryopreserved strains in repository (http://research.jax.org/grs/parkinsons.html). We developed simple material transfer agreements to better facilitate a timely distribution of animal models to PD researchers. This focused effort to get the best research tools into the hands of PD researchers reflects our position that animal models are crucial for the PD field.

## Discussion

In creating a standardization framework for animal model characterization, MJFF’s goal is to provide guidance to the PD research community as to the best practices for the use of animal models. This allows researchers to use the most appropriate models and measures, rather than spending time and effort creating their own standards. This also better positions the research community to conduct more specialized characterization of these models and to have tools that cater to different research interests.

Data from MJFF’s characterization efforts are being made available in multiple ways, including presentations at scientific meetings, which will ultimately result in formal publication. MJFF is also exploring ways to make high-quality images of immunohistochemistry from the 4-, 8- and 12-month-old animals available to the research community. In this way, MJFF invites researchers to draw independent conclusions from the data in order to build consensus about the most relevant and useful animal models. If successful, this would allow academia and industry to de-risk programs and provide a standardized framework to evaluate potential PD therapeutics.

Although our goal is to develop and distribute useful animal models and data to the PD research community as quickly as possible, there must be a balance between rapid dissemination of the tools and sufficient characterization to ensure quality assurance of the tools. This is a challenge because there might be occasions when PD researchers would like to obtain these animal models as quickly as possible, before the models have been rigorously characterized. Conversely, there might be occasions that PD researchers want fully characterized tools prior to them being made available. Therefore, our strategy has been to provide researchers with animal models that have gone through quality control (e.g. gene expression validation) and, in the meantime, carry out an in-depth characterization of the animal models.

The same systematic analysis of animal model testing could also be applied to therapeutic testing. Many researchers approach MJFF with potential therapeutics that they want tested in reliable animal models. Even though the Committee to Identify Neuroprotective Agents for Parkinson’s (CINAPS; also known as the Standardized Animal Model Screening of Neuroprotective Agents for Parkinson’s Disease) framework, developed by the National Institute of Neurological Disorders and Stroke, has been successfully utilized (De Jesús-Cortés et al., 2012), extending this framework for use with any PD therapeutic (from academia or industry) on MJFF-generated or -supported animal models would be useful. This standardized approach has also been employed in the amyotrophic lateral sclerosis (ALS) field. Therapeutic testing in ALS animal models has shown very different results depending on the design of the experiment. Rigorous experimental controls such as sibling matching, gender balancing, investigator blinding and transgene copy number verification might minimize the likelihood of attaining a false-positive therapeutic effect in SOD1G93A animal models of familial ALS (Gill et al., 2009). In systematically characterizing PD animal models, MJFF is laying the groundwork for which animal models ought to be used for preclinical therapeutic testing.

Despite MJFF and the research community’s best efforts, it is plausible that an animal model that fully recapitulates the cardinal features of PD will not be identified. Many transgenic animal models have failed to display PD-like phenotypes. MJFF is currently generating and evaluating transgenic models in multiple species and also exploring the viral vector approach of introducing pathogenic mutations *in vivo* to induce neurodegeneration in PD-relevant brain pathways. However, both of these approaches have proven to be challenging. It might be necessary to not only focus on generating an animal model of PD that has all of the facets of the PD phenotype but to determine the translatability of potential biomarkers that are expressed in animal models. These approaches are not mutually exclusive and, moreover, crucial information could be provided from an animal model that does not recapitulate the PD phenotype. Having a reliable, validated biomarker would be a major breakthrough in the PD field. A therapeutic window based on a biomarker that is closely linked to the genetic target and extensive safety evaluations might be sufficient to move a promising therapeutic forward to the clinic. The Alzheimer’s disease field is an example of a neurodegenerative disease that has established a robust biomarker (i.e. β-amyloid). With the absence of a biomarker in PD, it could be argued that the state of the PD field today is analogous to the Alzheimer’s field years ago. Therapeutics against Alzheimer’s disease can be prioritized based on the impact on biomarkers rather than relying solely on animal model cognitive data. We might have to move away from models of human PD and towards models of disease biology with the goal that they provide predictive validity in the clinic.

In the absence of a biomarker, animal models that do not exhibit PD-like phenotypes might still be useful owing to expression of the gene target in PD-relevant tissues. A transgenic and gene-targeted animal model (overexpression, knock-in and/or knockout) might play an important role in validating reagents such as antibodies and determining target engagement. The latter is extremely important because monitoring drug-target engagement facilitates clinical trials. For example, MJFF is developing both *SNCA* and *LRRK2* positron emission tomography (PET) tracers that require animal models to screen potential radiolabeled ligands. The establishment of receptor concentration (*B*_max_) and dissociation equilibrium constant (*K*_d_) values to evaluate the properties of potential PET tracers might also require transgenic animal models. *Ex vivo* binding assays utilizing tissues from well-characterized animal models would be crucial for screening radiolabeled ligands in order to optimize future PET tracers. In the absence of animal models with face validity, MJFF views these animal models as tools to establish target engagement. MJFF has taken a proactive role in generating, characterizing and distributing animal models of PD, and is also developing a best practices for use of PD animal models to promote more efficient use of these research tools. Future efforts will include systematically evaluating non-transgenic approaches (e.g. viral vectors), evaluating pharmacokinetic-pharmacodynamic relationships of promising preclinical drug candidates, and exploring other ways that animal models can be used to promote PD research and drug development. Although challenging, MJFF remains committed to providing the best tools to PD researchers both in academia and industry in order to accelerate a cure for those with PD.
